# Orthoplastics in Periprosthetic Joint Infection of the Knee: Treatment Concept for Composite Soft-tissue Defect with Extensor Apparatus Deficiency

**DOI:** 10.7150/jbji.47018

**Published:** 2020-05-18

**Authors:** Rik Osinga, Maurice Michel Eggimann, Steven John Lo, Richard Kühl, Alexander Lunger, Peter Emil Ochsner, Parham Sendi, Martin Clauss, Dirk Johannes Schaefer

**Affiliations:** 1 Centre for Musculoskeletal Infections, University Hospital Basel, Basel, Switzerland; 2 Department of Plastic, Reconstructive, Aesthetic and Hand Surgery, University Hospital Basel, Basel, Switzerland; 3 Canniesburn Plastic Surgery Unit, Glasgow Royal Infirmary, 84 Castle Street, Glasgow, G4 0SF, United Kingdom; 4 Translational Research Center, Kaohsiung Medical University Hospital, Kaohsiung Medical University, Kaohsiung, Taiwan; 5 Infectious Diseases and Hospital Epidemiology, University Hospital Basel, Basel, Switzerland; 6 Clinic for Orthopedics and Trauma Surgery and Interdisciplinary Septic Surgical Unit, Kantonsspital Baselland, Liestal, Switzerland; 7 Institute for Infectious Diseases, University of Bern, Bern, Switzerland; 8 Department of Orthopaedic and Trauma Surgery, University Hospital Basel, Basel, Switzerland

**Keywords:** Knee, TKA, PJI, infected arthroplasty, soft-tissue defect, deficient extensor apparatus, orthoplastic, surgical concept

## Abstract

**Introduction**: Reconstruction of composite soft-tissue defects with extensor apparatus deficiency in patients with periprosthetic joint infection (PJI) of the knee is challenging. We present a single-centre multidisciplinary orthoplastic treatment concept based on a retrospective outcome analysis over 20 years.

**Methods and Results**: One-hundred sixty patients had PJI after total knee arthroplasty. Plastic surgical reconstruction of a concomitant perigenicular soft-tissue defect was indicated in 47 patients. Of these, six presented with extensor apparatus deficiency. One patient underwent primary arthrodesis and five patients underwent reconstruction of the extensor apparatus. The principle to reconstruct missing tissue 'like with like' was thereby favoured: Two patients with a wide soft-tissue defect received a free anterolateral thigh flap with fascia lata; one patient with a smaller soft-tissue defect received a free sensate, extended lateral arm flap with triceps tendon; and two patients who did not qualify for free flap surgery received a pedicled medial sural artery perforator gastrocnemius flap. Despite good functional results 1 year later, long-term follow-up revealed that two patients had to undergo arthrodesis because of recurrent infection and one patient was lost to follow-up.

**Conclusion**: These results show that PJI of the knee and extensor apparatus deficiency is a dreaded combination with a poor long-term outcome. Standardization of surgical techniques for a defined PJI problem and consensus on study variables may facilitate interinstitutional comparisons of outcome data, and hence, improvement of treatment concepts.

## Introduction

In patients with periprosthetic joint infection (PJI) after total knee arthroplasty (TKA) and a concomitant soft-tissue defect, the extensor apparatus can be deficient. This presents a significant challenge for orthoplastic reconstruction, and a multidisciplinary strategy - including a thoroughly planned orthoplastic approach - is imperative. The lack of an orthoplastic concept may lead to further soft-tissue damage with bone stock loss and, eventually, to limb amputation. Multiple comorbidities contribute to host-related risk factors, aggravating the potential for failure 1. These considerations underline the importance of referring these patients early to a specialized multidisciplinary bone and joint infection (BJI) unit 2-4. However, most orthoplastic concepts are published on chronic long bone osteomyelitis 5. Here, we present a single-centre multidisciplinary orthoplastic treatment concept based on a retrospective outcome analysis over 20 years. The technical approach for concomitant soft-tissue and extensor apparatus reconstruction is highlighted and the available literature on this topic discussed.

## Methods

### Patient Selection Workup

A prospectively maintained database of patients treated at the University Hospital of Basel from 1999 until 2020 was retrospectively searched for patients with PJI after TKA and concomitant soft-tissue defects with extensor apparatus deficiency. The presence and extent of extensor apparatus deficiency was intraoperatively defined by the orthopaedic surgeon.

### Clinical Case Analysis

(i) What was the indication for plastic surgery?

(ii) Which plastic surgical techniques were used to reconstruct both the outer soft-tissue envelope and the underlying deficient extensor apparatus of the knee?

(iii) When were the plastic surgical procedures performed with regard to the orthopaedic treatment concept?

(iv) What was the orthoplastic long-term outcome?

### Ethics Committee Approval and Patient Consent

The study was conducted according to legal regulations of the Swiss Human Research Act and approved by the local ethical committee (EKNZ 2019-00265). All included patients gave consent to use their health-related data.

### Literature Search

**Figure 1:**
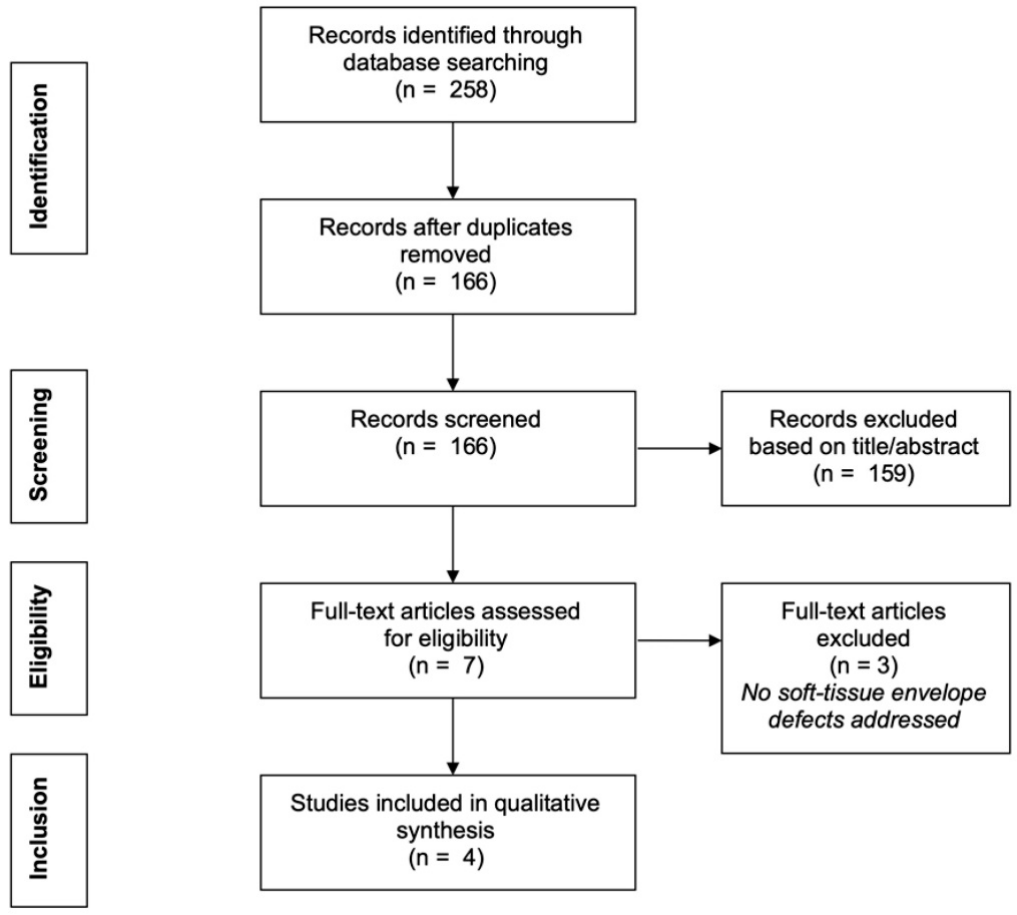
Literature search flow diagram.

In order to identify relevant articles to address these questions, in accordance with the PRISMA guidelines, we undertook a systematic search of the PubMed database for articles from January 1966 until March 2020 with the following predetermined inclusion criteria (Figure 1): (i) published reports on the treatment of deficient extensor apparatus of the knee in patients with PJI after TKA and soft-tissue envelope defects, (ii) all levels of evidence, and (iii) all languages included. Exclusion criteria were reports on (i) extensor apparatus deficiency with an underlying cause other than PJI (e.g. neoplasm, trauma), (ii) treatment of TKA with PJI and a soft-tissue defect that did not specifically address treatment of the deficient extensor apparatus, and (iii) treatment of extensor apparatus deficiency in patients without PJI. Major Medical Subject Heading (MeSH) terms were identified and additional search terms used to narrow the search, using the following six combinations (alternative terms grouped in parentheses): (1) 'Prosthesis-Related Infections' [MeSH] AND ('Extensor' OR 'Extensor Apparatus' OR 'Patellar Tendon'), (2) ('Extensor Mechanism' OR 'Extensor Apparatus') AND 'reconstruction' AND 'infection', (3) 'Treatment' AND ('Periprosthetic Joint Infection' OR 'Implant Associated Infection' OR 'Prosthesis Infection' OR 'Arthroplasty Infection') AND ('Extensor Mechanism' OR 'Extensor Apparatus' OR 'Patellar Tendon'), (4) 'soft tissue defect*' AND 'reconstruction*' AND 'tendon*' AND 'knee*', (5) 'knee' AND ('extensor mechanism' OR 'extensor apparatus') AND 'infection*' AND 'reconstruction*', and (6) 'knee' AND 'tendon* replacement'.

## Results

### Patient Selection Workup

**Figure 2:**
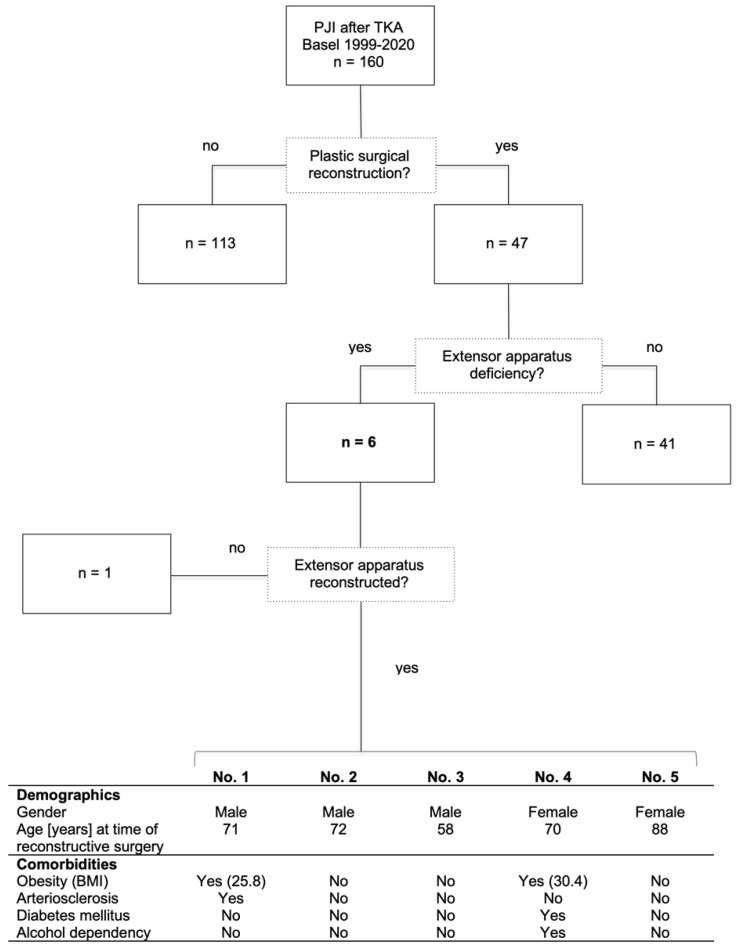
Inclusion criteria and patient demographics. PJI: periprosthetic joint infection; TKA: total knee joint arthroplasty; BMI: body mass index [kg/m^2^].

At the University Hospital Basel, 160 patients were treated for PJI after TKA between 1999 and March 2020 (Figure 2). Plastic surgical reconstruction of a perigenicular soft-tissue defect was indicated in 47 patients. Of these, six had extensor apparatus deficiency. One patient underwent primary arthrodesis, as reconstructive surgery posed too great a risk for the patient's morbidity and mortality. Five patients (three male, two female) underwent knee extensor apparatus reconstruction at an average age of 72 (58-88) years at the time of reconstructive surgery. Three had no comorbidities; one was overweight (body mass index [BMI] 25.8) and arteriosclerotic; and one had obesity (BMI 30.4), diabetes, and alcohol dependency.

**Figure 3:**
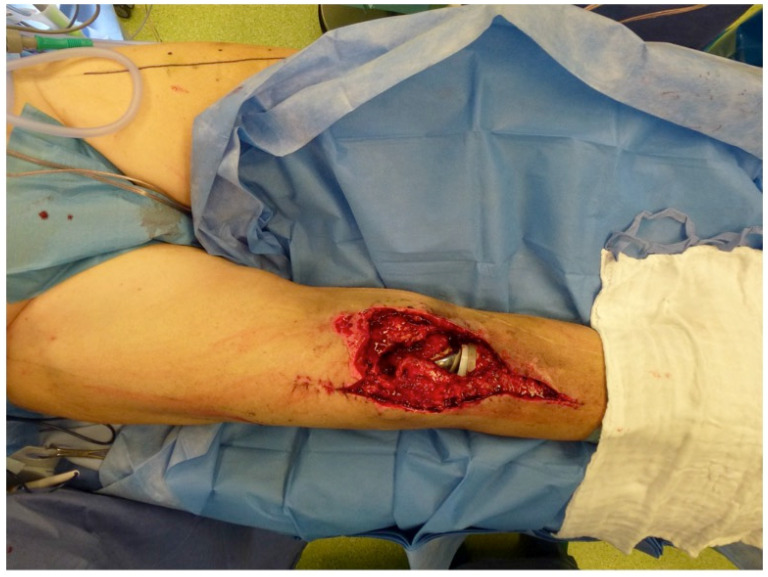
Partial defect of the extensor apparatus (EA): Right knee of a patient with PJI after TKA and a debridement, antibiotics and implant retention procedure, and a two-stage procedure performed twice. The combination of a medial and lateral incision pattern led to a large postoperative soft-tissue necrosis with a concomitant 50% patellar tendon and 40% quadriceps tendon lesion (Table 1: Patient No. 1; additional Figures 10, 11).

**Figure 4:**
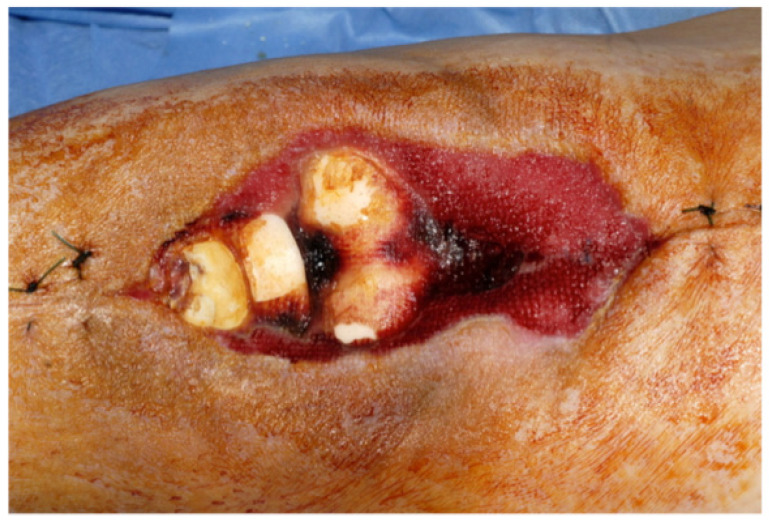
Total loss of the EA: Chronically infected TKA after several debridements, spacer implantation, and negative pressure wound therapy (NPWT) (Table 1: Patient No. 2).

**Figure 5:**
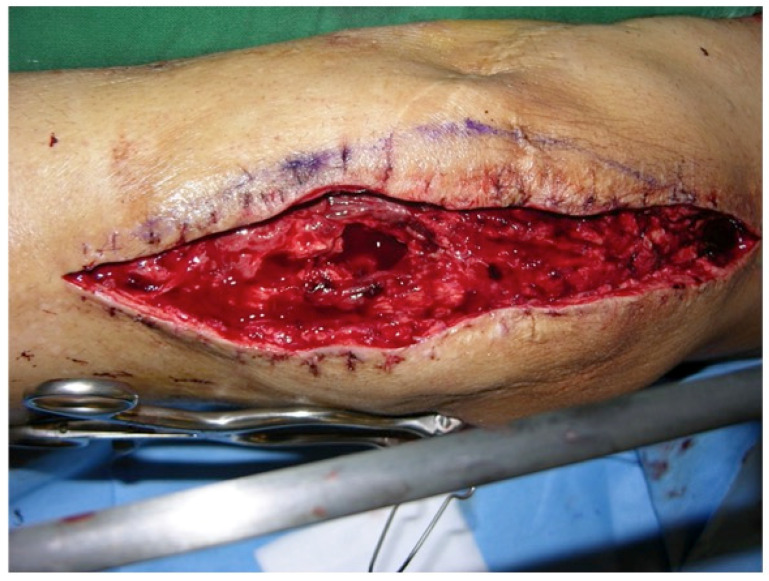
Partial loss of the EA: PJI of the right knee with a 70% patellar tendon lesion after secondary TKA in an area with excessive scarring. Previously, this patient had undergone 20 operations because of chronic osteomyelitis after a complex comminuted fracture of the right femoral shaft after being hit by a car as an adolescent. The TKA was removed followed by debridement, application of an external fixator, and soft-tissue reconstruction with a free sensate upper lateral arm flap before TKA reimplantation seven weeks later (Table 1: Patient No. 3; additional Figures 7, 8, 9, 18, and 19).

**Figure 6:**
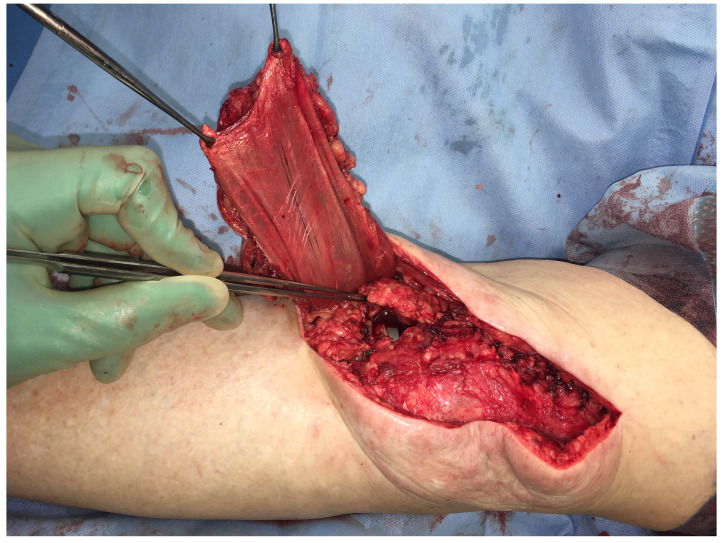
Pedicled MSAP gastrocnemius flap: Left knee with combined 40% patellar tendon and medial capsule lesion during the second stage of a two-stage procedure. Initial wound dehiscence after primary TKA had been treated with NPWT. Note the tendinous back of the medial gastrocnemius muscle already in place for medial capsule/patellar tendon strengthening (Table 1: Patient No. 5; additional Figures 12, 13, 14, 15, 16, 17).

The extent of damage to the extensor apparatus varied: One patient had a combined 50% patellar and 40% quadriceps tendon lesion (Figure 3); one had complete loss of the patellar tendon, patella bone, and quadriceps tendon (Figure 4); two had a 70% patellar tendon lesion (Figure 5); and one had a 40% patellar tendon lesion (Figure 6) after debridement. The median number of operations on the affected knee (joint aspirations excluded) before orthoplastic reconstruction was 8 (3-20) (Table 1).

### Indication for Plastic Surgery

The condition of the perigenicular soft tissue dictated the indication for plastic surgery. In general, any soft-tissue envelope around the infected TKA that did not allow tension-free direct closure was an indication for plastic surgery involvement. In the five patients with a deficient knee extensor apparatus who underwent reconstructive surgery, chronic inflammation was implied and extensive soft-tissue damage found. Thus, there was an indication for plastic surgery involvement.

### Plastic Surgical Techniques

Thorough preoperative analysis of the soft-tissue defect defined the damaged structures to be reconstructed. These structures usually consisted of the following tissue types: (i) skin, (ii) hypodermis, and (iii) tendon. The principle to reconstruct 'like with like' was followed: Vascularized autologous tissue replaced skin with cutis, hypodermis with subcutaneous tissue, and tendon with tendinous tissue. The choice of flap depended both on the preoperative analysis of the soft-tissue defect and on the individual patient's characteristics. The three techniques performed that exemplify these principles are outlined below.

#### Free Sensate Extended Lateral Arm Flap (s-ELA) with Triceps Tendon (TT) (Figure 7)

*Indication*: Small soft-tissue defect, within the limits of primary closure of the donor site (up to ~7 cm width), and a partial patellar or quadriceps tendon defect.

**Figure 8:**
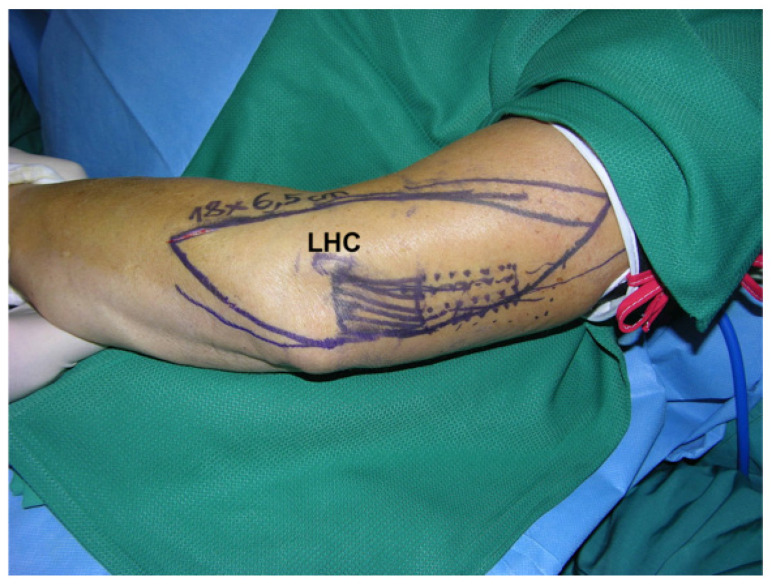
Free s-ELA: Markings to raise the flap with a triceps tendon (18 x 6.5 cm dimension) over the lateral humeral condyle (LHC) (Table 1: Patient No. 3; additional Figures 5, 7, 9, 18, 19).

**Figure 9:**
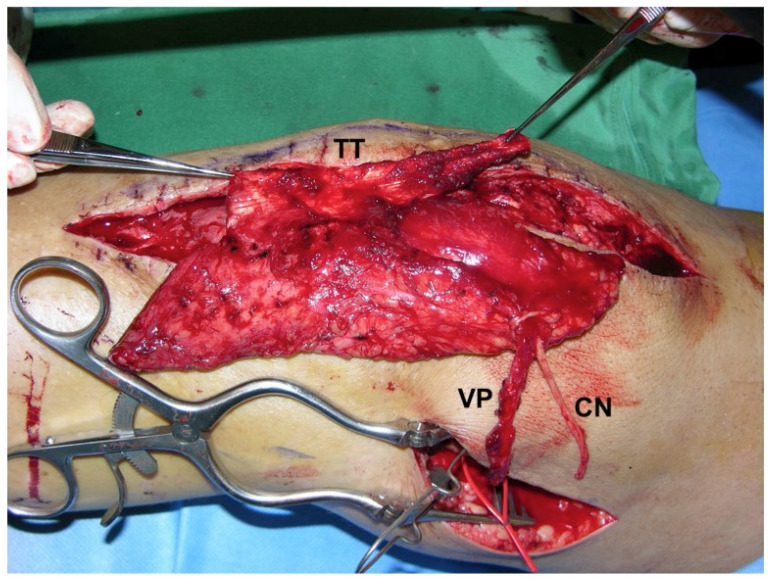
Free s-ELA: Provisional inset of the flap with triceps tendon (TT) held by forceps to reconstruct the patellar tendon defect. An additional incision was needed for microsurgical anastomosis of the vascular pedicle (VP) of the flap to the descending genicular vessels and epineural nerve coaptation of the cutaneous nerve (CN) of the flap to the saphenous nerve (Table 1: Patient No. 3; additional Figures 5, 7, 8, 18, 19).

*Surgical technique*: This procedure was first described by Song et al. 6, and later as an extended lateral arm flap by Kuek and Chuan 7. The patient is positioned supine and the donor arm placed on a hand table. A line is drawn between the deltoid tuberosity and the lateral humeral condyle, marking the lateral intermuscular septum over the radial collateral artery. If the defect is long, the flap can be extended to the forearm (Figure 8). Dissection is usually from an anterolateral approach and deepened to the triceps muscle fascia. This approach allows visualization of the intermuscular septum. Up to one third of the width of the distal triceps tendon with parts of the lateral head can be included without functional loss. During dissection underneath the triceps tendon, it remains attached to the fascia of the flap because it is vascularized by the prefascial vascular plexus. The flap is elevated towards the septum, including the deep muscular fascia. Flap raising is continued in a retrograde manner. The posterior cutaneous nerve of the forearm may need to be sacrificed, which results in hyposensation in a small area on the proximal dorsal forearm. However, for free flap sensation, the inferior lateral cutaneous nerve of the arm 8 can be preserved and used for coaptation to the saphenous nerve. To gain maximum pedicle length, we dissect the vascular bundle until it branches off the radial collateral artery, carefully protecting the radial nerve to avoid postoperative neurapraxia. The composite flap is provisionally set into the defect and microvascular anastomoses are performed. This technique often requires a separate incision to reach the nearest donor vessel (Figure 9). The triceps tendon is then used to reconstruct the patellar or quadriceps tendon.

#### Free Anterolateral Thigh (ALT) Flap with Fascia Lata (FL) (Figure 10)

*Indication:* Wider soft-tissue defect, within the limits of primary closure of the donor site (up to ~10 cm width), and an extended or complete patellar or quadriceps tendon defect.

**Figure 11:**
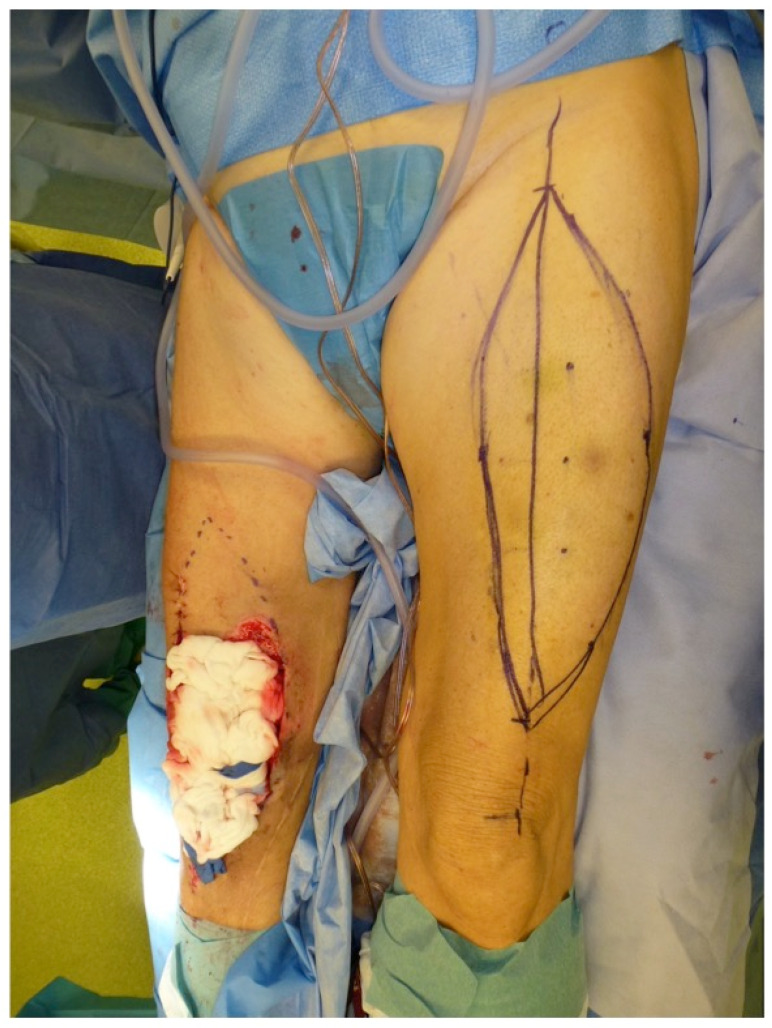
Free ALT with FL: Intraoperative markings to raise the flap to cover the defect around the right knee. The marked dots indicate the skin perforators (identified with a Doppler) off the descending branch of the lateral femoral circumflex artery. Note the planned resection of intact skin proximal to the defect of the right knee to allow flap inset (Table 1: Patient No. 1; additional Figures 3, 10).

*Surgical technique:* The procedure we use is consistent with previous descriptions 9, 10. In brief, a line is drawn on the donor leg with the foot in a strictly supine position between the anterior superior iliac spine and the upper lateral border of the patella. The skin perforators are marked around the midpoint of that line with the use of a handheld Doppler. The provisional flap design is centred around the perforator markings (Figure 11). The flap is raised through a medial incision over the rectus femoris muscle and deepened subfascially to verify the perforator anatomy. The skin flap design can then be finalized around the visualized perforators off the descending branch of the lateral femoral circumflex artery. Dissection is extended laterally to the vastus lateralis muscle to include the adjoining fascia lata. Together with an adequate fascia lata portion, the flap is elevated as a composite flap. The portion of fascia lata raised corresponds to the extent of the tendinous defect. Hence, the composite flap includes the subfascial and prefascial vascular plexus, which vascularizes the fascia 9. The composite flap is provisionally set into the defect and the microvascular anastomoses are then performed. The folded fascia lata is used to reinforce the patellar or the quadriceps tendon or both. The skin is closed in a multilayer fashion after drain placement.

#### Pedicled Medial Sural Artery Perforator (MSAP) - Gastrocnemius Flap (Figure 12)

*Indication:* Soft-tissue defect and a partial patellar or quadriceps tendon defect in patients who do not qualify for free flap surgery.

**Figure 12:**
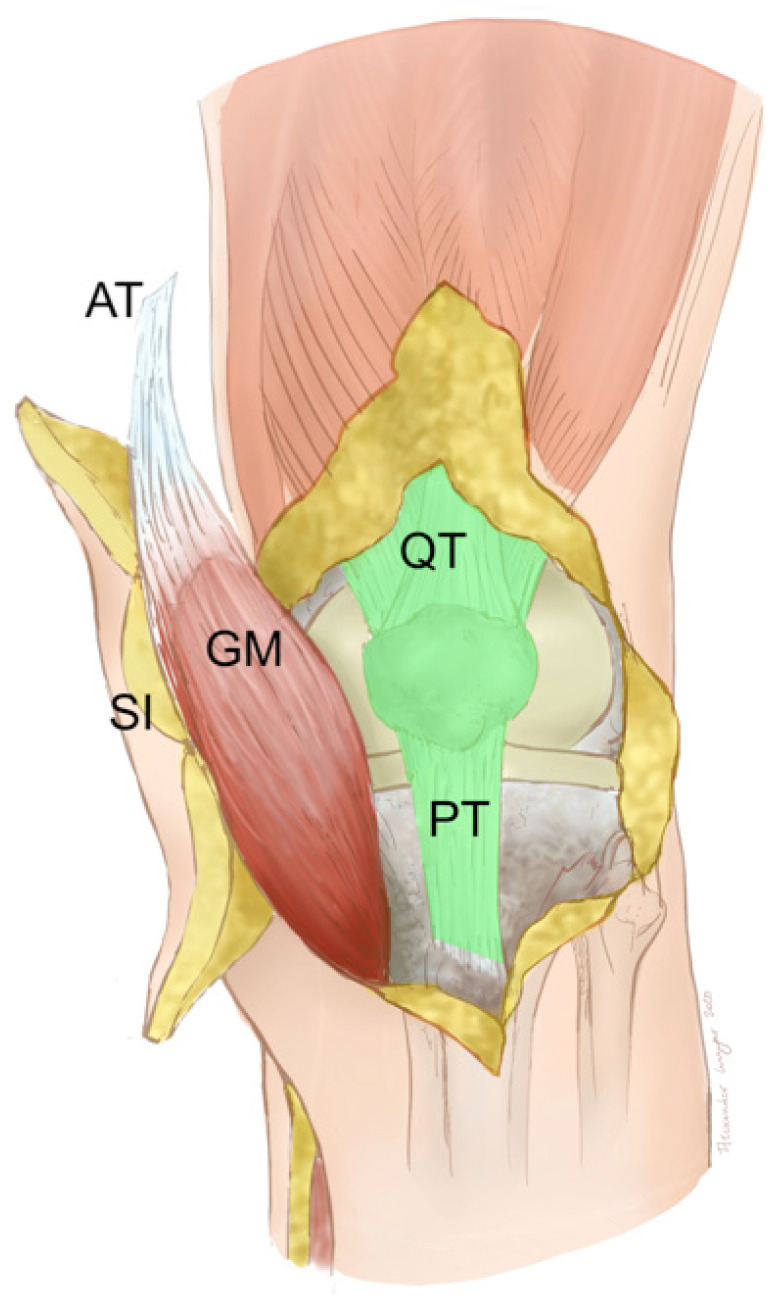
Pedicled MSAP gastrocnemius flap: Clinical scenario of PJI after TKA in a patient who does not qualify for free flap surgery with a soft-tissue defect and an underlying extensor apparatus deficiency with either a partial patellar tendon (PT) lesion or partial quadriceps tendon (QT) lesion (light green). The soft-tissue defect is reconstructed with a MSAP gastrocnemius flap. Shown is the situation after the flap has been guided underneath the intact skin bridge that contains the saphenous nerve and vein. The medial gastrocnemius muscle (GM) has been raised with part of the Achilles tendon (AT) and the overlying skin island (SI) fed by the MSAP (Table 1: Patient No. 5; additional Figures 6, 13, 14, 15, 16, 17).

**Figure 13:**
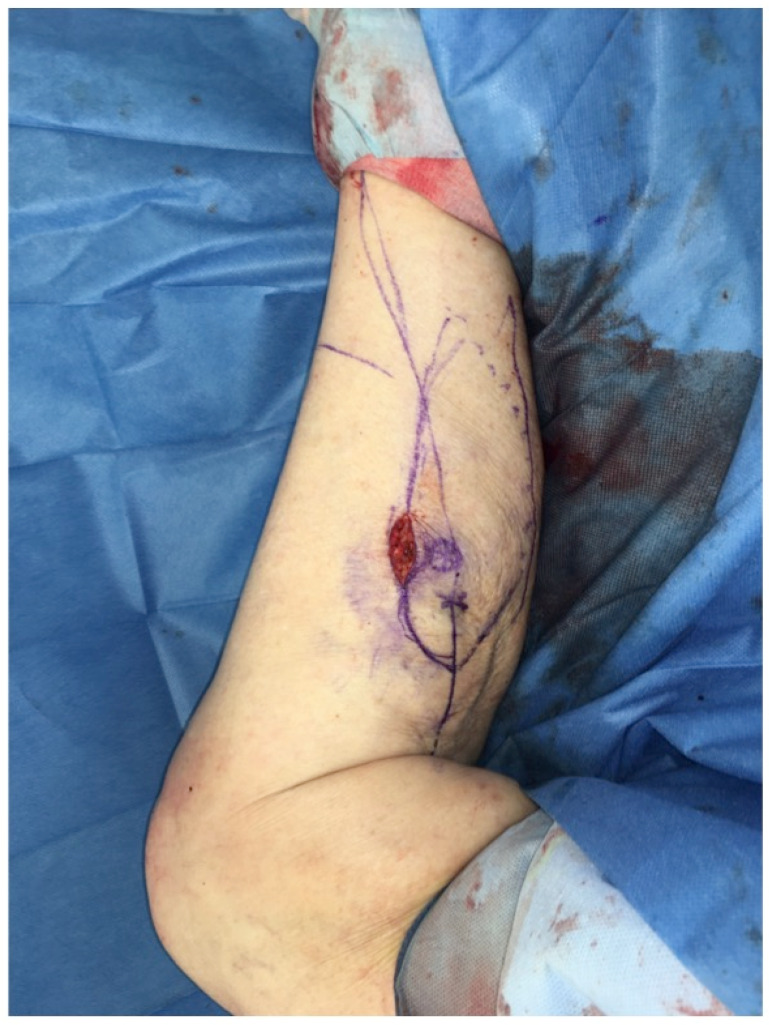
Pedicled MSAP gastrocnemius flap: Intraoperative drawing to raise the flap. The crosses indicate the skin perforators (identified with a Doppler) over the medial gastrocnemius muscle. An anterior incision is performed to visualize and verify the perforator anatomy. The skin island is then designed around the perforators according to the soft-tissue defect (Table 1: Patient No. 5; additional Figures 6, 12, 14, 15, 16, 17).

**Figure 14:**
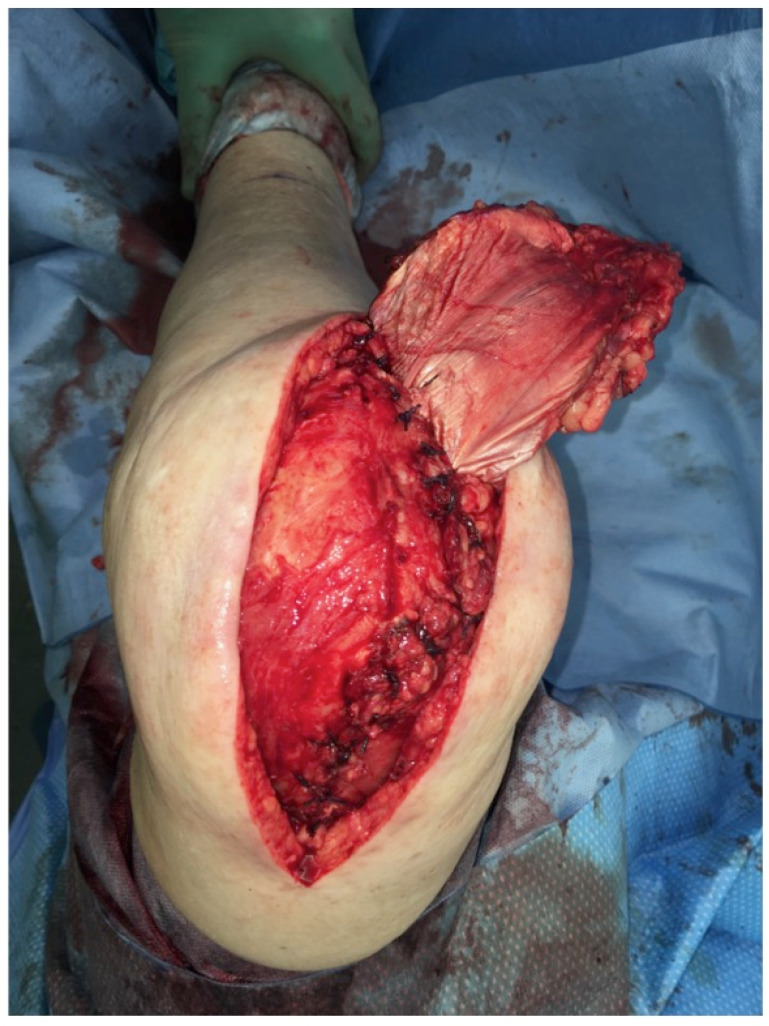
Pedicled MSAP gastrocnemius flap: Left knee in flexion while strengthening the medial capsule/patellar tendon lesion with the tendinous dorsal part of the flap (Table 1: Patient No. 5; additional Figures 6, 12, 13, 15, 16, 17).

**Figure 15:**
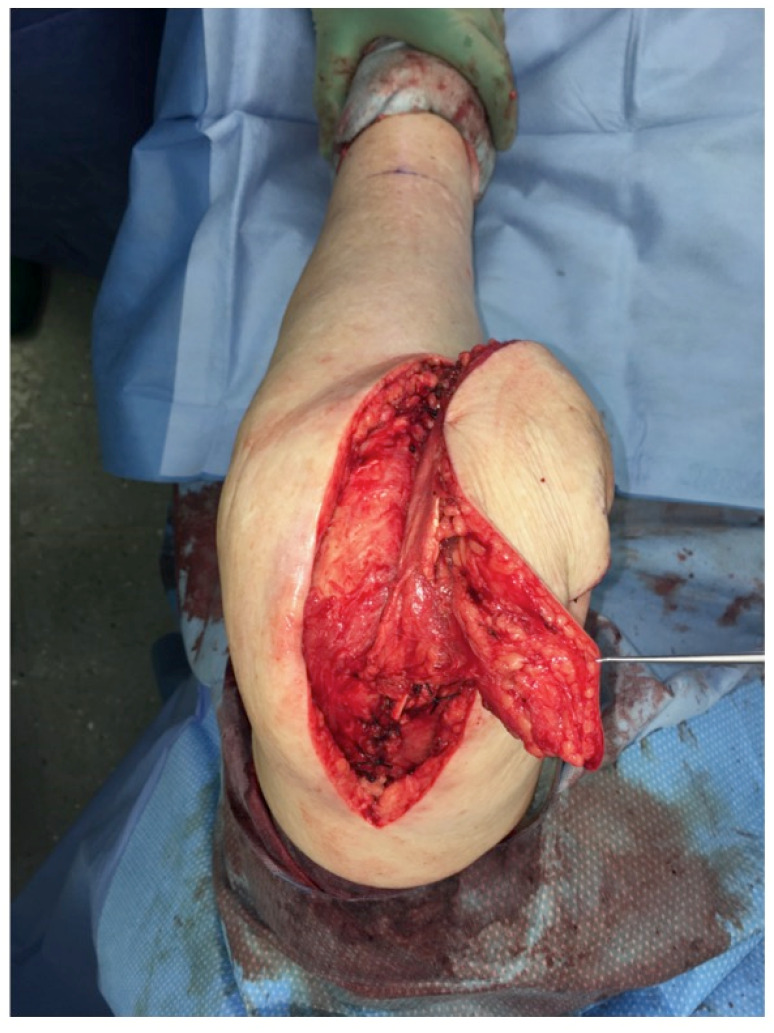
Pedicled MSAP gastrocnemius flap: The tendinous part of the flap is continuously sutured over the medial arthrotomy incision while holding the knee in a flexed position (Table 1: Patient No. 5; additional Figures 6, 12, 13, 14, 16, 17).

**Figure 16:**
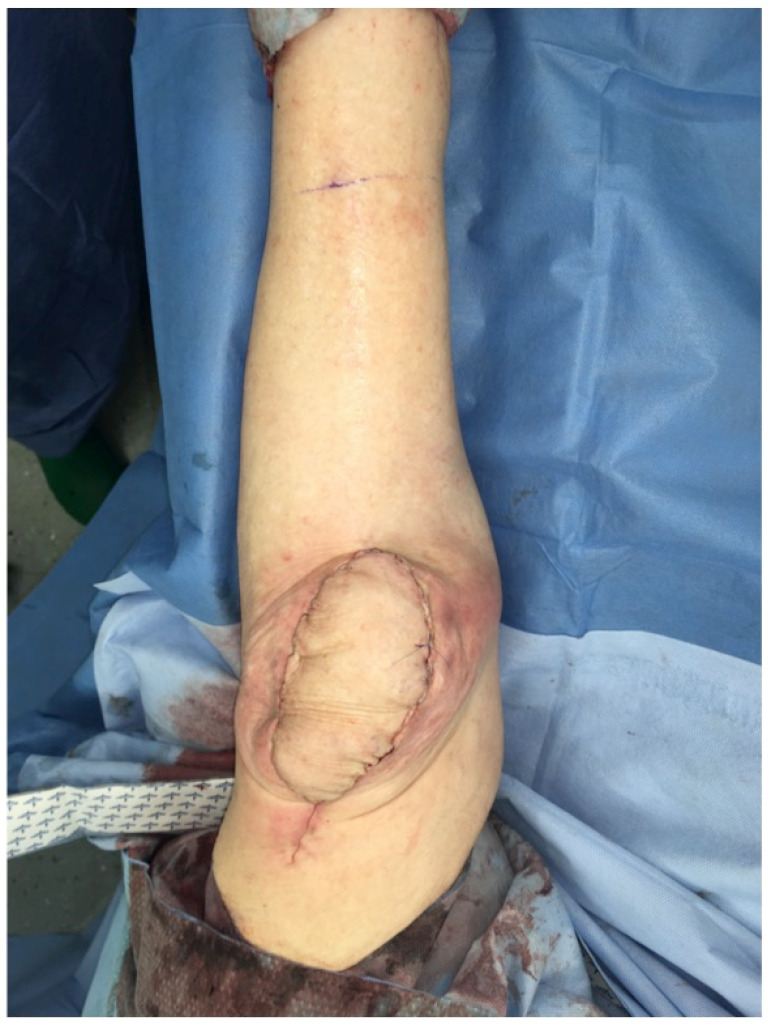
Pedicled MSAP gastrocnemius flap: Intraoperative view with knee in full extension after soft-tissue inset during flexion to avoid stress on the soft tissue during mobilization (Table 1: Patient No. 5; additional Figures 6, 12, 13, 14, 15, 17).

**Figure 17:**
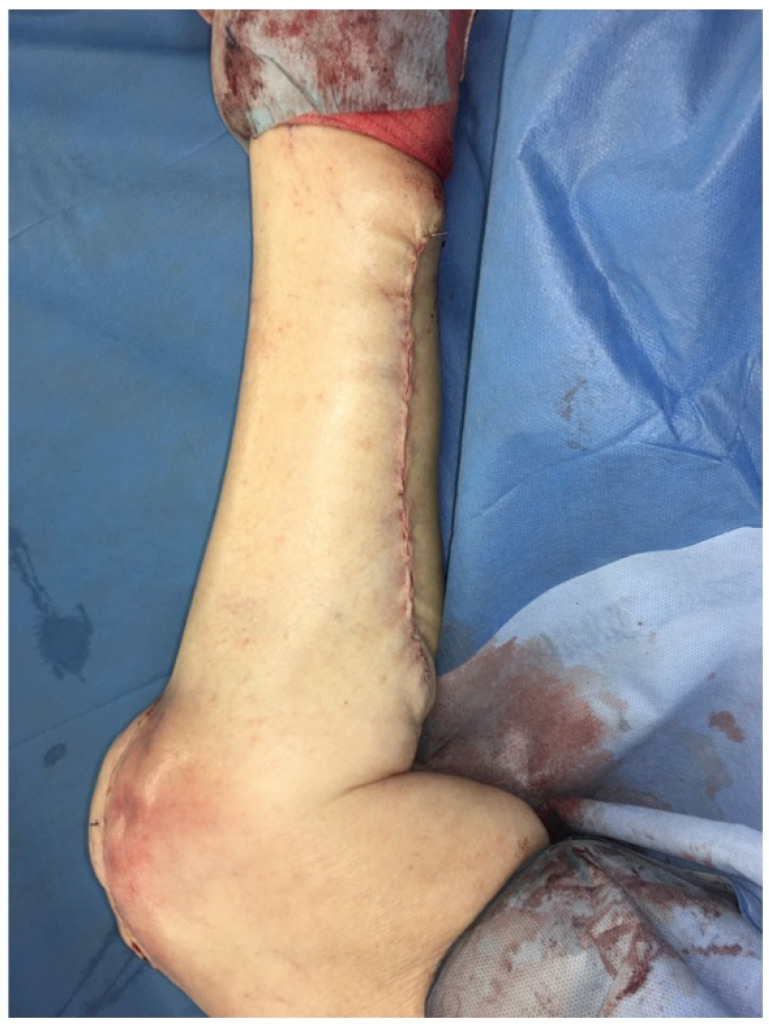
Pedicled MSAP gastrocnemius flap: Intraoperative view of the medial left knee after direct closure of the donor site. The flap integrates nicely into the defect and shows little bulk, which will diminish over time, as the muscle will atrophy significantly. In elderly patients, the laxity of the posterior skin of the calf allows direct closure of the donor site more easily (Table 1: Patient No. 5; additional Figures 6, 12, 13, 14, 15, 16).

*Surgical technique:* The medial sural artery supplies the medial gastrocnemius muscle and sends perforating branches to the skin. These MSAPs are sometimes tortuous and do not always directly correlate with the Doppler signal 11, 12. A line is drawn between the midpoint of the popliteal crease and the medial malleolus. Perforators are found between 8 and 15 cm distal to the popliteal crease, although anatomical variation is common 13. A handheld acoustic Doppler verifies the perforator vessels. Dissection approaches from the anterior with subfascial verification of the perforator(s) anatomy (Figure 13). The MSAP component of the combined flap can be isolated on the main perforator(s) to maximize independent movement of the fasciocutaneous and muscular components of the flap. The fasciocutaneous component is placed vertically for external skin cover and the muscle positioned obliquely or horizontally for knee capsule reconstruction. The medial gastrocnemius muscle is raised with preservation of the sural nerve. The strong tendinous dorsal aspect of the muscle can be used for partial patellar tendon defect reconstruction or knee capsula reinforcement (Figures 14,15). The musculocutaneous flap is raised far proximally until it can easily reach the soft-tissue defect. It may be necessary to detach the flap off the medial condyle of the femur. Flap insetting is ideally performed with the knee in 90° flexion to avoid stress on the soft tissue (Figure 16) during knee mobilization. The donor site can often be closed directly (Figure 17), provided that the fasciocutaneous part of the flap does not exceed a width of ~6 cm 14. If the defect of the extensor apparatus requires reconstruction of the quadriceps tendon, the flap can be raised with part of the Achilles tendon (Figure 12).

After reconstructive surgery, all patients remained in bed with a knee brace in full extension for 5 days before flap training started. The knee brace was applied for 8 weeks and full weightbearing from week 2 on was allowed. Thereafter, gradual continuous passive motion of the knee joint started under physiotherapeutic instruction and supervision.

### Timing of Plastic Surgery

The combined soft-tissue and extensor apparatus reconstruction took place during a one-stage procedure in one patient, within the 6-week interval of a two-stage procedure in two patients, during the second stage in one patient, and after the second stage in one patient.

### Orthoplastic long-term outcome

**Figure 18:**
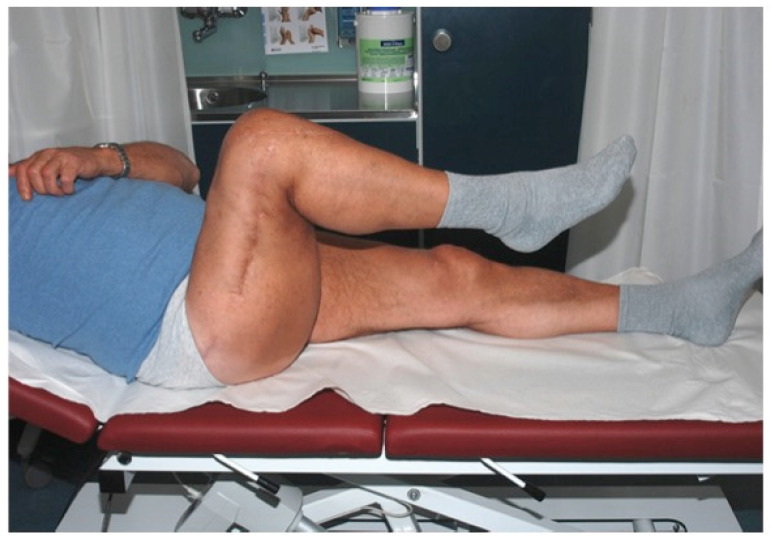
Free s-ELA: Lateral view of the right knee showing excellent flexion 12 months after concomitant soft-tissue and patellar tendon defect reconstruction (Table 1: Patient No. 3; additional Figures 5, 7, 8, 9, 19).

**Figure 19:**
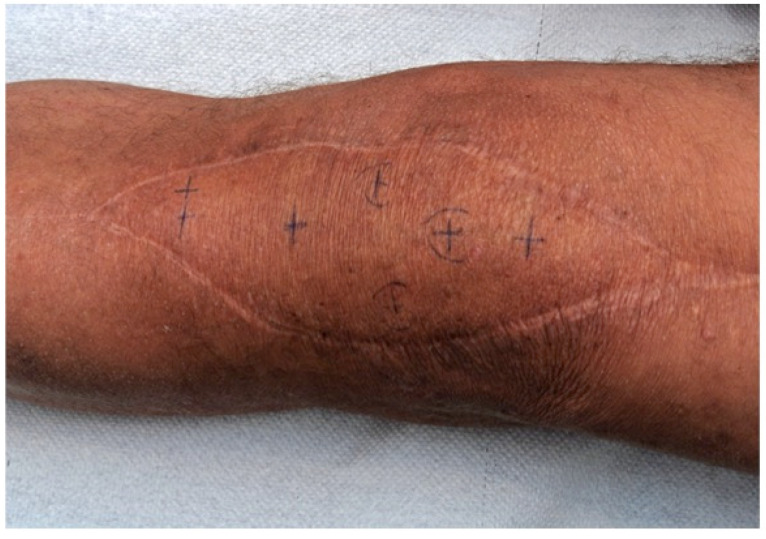
Free s-ELA: Anterior view of the right knee after extended lateral arm flap showing excellent sensation medially and intermediate reinnervation laterally after 12 months (Table 1: Patient No. 3; additional Figures 5, 7, 8, 9, 18).

Reconstruction of the extensor apparatus was successful in all cases and no wound healing problems were observed. The flap condition was favourable in all patients, with one patient complaining of minor swelling. One year postoperatively, knee function (active extension/flexion) was reported as follows (Table 1): free ALT flap with FL 0/0/80° and 0/5/100°; free s-ELA flap with TT 0/0/100° (Figure 18). One patient has not yet completed the 1-year follow-up (No. 5) and one patient was lost to follow-up (No. 2). In addition, the patient with the free s-ELA flap with TT (No. 3) reported very good sensation throughout the whole flap territory (Figure 19), a detailed case discussion can be found here 15. The average follow-up time was 3.5 (0.2-8) years after reconstructive surgery and revealed that two patients (No. 1 and 4) had undergone knee arthrodesis in our institution due to recurrence of PJI at 1.8 and 2 years after flap reconstruction (Table 1).

### Review of the Literature

The initial search identified 166 publications after removal of duplicates (Figure 1). Titles and abstracts were screened to identify applicable articles. In addition, reference lists of the identified articles were tracked for additional previously unidentified articles. This resulted in seven articles 10, 16-21 of which three 17-19 were excluded, as they elaborated on cases with PJI after TKA and extensor apparatus deficiencies but failed to include concomitant soft-tissue envelope defects. The remaining four articles 10, 16, 20, 21 included a total of four patients. It appeared likely that the described orthoplastic techniques were performed by a team of orthopaedic and plastic surgeons together, although this was not explicitly stated.

### Plastic Surgical Techniques and Orthoplasic Outcome

Chiou et al. used a pedicled lateral gastrocnemius muscle with attached lateral Achilles tendon for reconstruction 20. The muscle with the skin graft replaced the skin and hypodermis, and the Achilles tendon replaced the patellar tendon. This method resulted in an active range of motion (extension/flexion) of 0-10-75° after 18 months.

Pérez-García et al. used a myocutaneous MSAP gastrocnemius flap to reconstruct the skin and hypodermis and a pedicled gracilis and semitendinosus tendon to reconstruct the patellar tendon 16. An active range of motion (extension/flexion) of 0-0-100° was reported after 12 months.

Sapino et al. published two cases of defect reconstruction with a free composite ALT flap, including the vastus lateralis and FL 10. Di Summa et al. performed a retrospective functional investigation of 21 ALT flaps 21, investigating the same two patients in different contexts. The fasciocutaneous part of the flap reconstructed the skin and hypodermis defect, and the vascularized fascia lata was used to reconstruct the patellar tendon defect. An active range of motion of 100° and 80° was reported after 18 months.

### Timing of Plastic Surgery

The curative orthopaedic treatment concept for chronic PJI of the knee frequently differentiates between a one-stage and a two-stage approach. For the combined orthoplastic approach, Chiou et al., di Summa et al., and Sapino et al. described a one-stage approach 10, 20, 21, whereas Pérez-García et al. reconstructed the soft-tissue envelope and extensor apparatus at the first stage of a two-stage approach 16. Despite these differences in stages, all institutions reported that they aimed to reconstruct the soft-tissue defect as early as possible.

## Discussion

The orthopaedic treatment algorithm for PJI has been previously well described 22, 23. Recently, an orthoplastic guide to the generalized management of complex joint reconstruction has been proposed 3. The combined multidisciplinary approach described herein is particularly important in patients with soft-tissue defects and extensor apparatus deficiency around the knee. We stress the importance of treating these patients in a specialized BJI unit to ensure the best possible outcome 3, 24. The interaction between various specialists as part of an orthoplastic treatment concept allows a simultaneous multidisciplinary approach while the patient is located in one institution. Although no studies have compared outcomes of patients treated in specialized BJI units and non-specialized centres, the variables associated with free flap failure in the context of lower limb reconstruction indicate chronic disease history with multiple interventions that are often performed at different centres. These variables include diabetes 25, 26, multiple comorbidities (diabetes, renal failure, and vascular disease) 27, chronic ulceration around the foot and ankle 26, and chronic osteomyelitis 27, emphasizing that these patients have to be thoroughly tested prior to adequate surgery.

In our opinion, any soft-tissue envelope around a TKA that does not allow tension-free direct closure should be an indication for plastic surgery involvement. We are convinced that the need for soft-tissue coverage must be recognized early and therefore patients must be referred early. Traditionally, plastic surgeons have been involved in treatment concepts for trauma patients that prioritize durable soft-tissue coverage over implants (e.g. reconstructing soft-tissue defects after open fractures). In these cases, the functional aspect played a subordinate role. In orthopaedic patients with PJI after TKA, however, a subpopulation has composite soft-tissue defects. Consequently, plastic surgeons are faced with new technical challenges. Soft-tissue defect reconstruction must not only provide stable and durable coverage, but it must also adequately address functional restoration of the underlying extensor apparatus. The plastic surgeon harvests the flap, including tendinous material, and performs the insertion into the defect, followed by the microvascular anastomoses. The orthopaedic surgeon then integrates the transplanted tendinous tissue to reconstruct the deficient extensor apparatus. We recommend reconstructing the soft-tissue defect during the first stage of a two-stage orthopaedic treatment concept. The rationale for this recommendation relies on the following arguments: Firstly, early surgery maximizes the time for the soft tissue to heal and integrate. Specifically, 8 weeks of protection time are needed after repair of a fully interrupted extensor apparatus. Secondly, a well-vascularized reconstructed tissue can act as a vehicle for the transport of antimicrobial agents to the site of infection. Thirdly, the smaller the number of interventions, the lower the rate of complications and number of anaesthetic procedures. Although these arguments have not yet been confirmed in well-designed scientific studies, our recommendation is congruent with the proposed timing for soft-tissue management in a retrospective analysis of 112 complex knee joint revision arthroplasties 3. Prospective studies from various institutions, possibly with different treatment approaches, are needed to further advance knowledge in this field.

In reality, however, patients are not always referred early, and adequate multidisciplinary treatment is sometimes delayed. This may lead to suboptimal results, as our small series shows: Two of five patients had to undergo knee arthrodesis because of recurrent infection despite encouraging results after 1 year. Clearly, the small number of patients included in our study cannot provide any statistical evidence, but the failures due to recurrent infection occurred in the two patients with the most comorbidities. Furthermore, none of our five patients were treated according to the timeline concept outlined earlier. One patient was treated in a one-stage procedure despite the presence of a sinus tract. In retrospect, a two-stage procedure should have been favoured. In the four patients undergoing a two-stage procedure, soft-tissue reconstruction did not take place during the first of two stages. The detailed reasons for not performing the plastic reconstruction early in these cases remain speculative. Timely coordination of all specialities in one single procedure is still challenging in daily clinical practice. The psychological burden for patients to consent to another intervention might also contribute to the delay.

Clearly, our patients endured much, as on average they had undergone eight operations before flap surgery. But even in patients without a soft-tissue defect of the perigenicular envelope, PJI and extensor apparatus deficiency is a dreaded combination with a poor outcome. In a recent multicentre study of patients with PJI and extensor apparatus reconstruction, only 23% were successful and 77% considered failures with recurrence of infection 18.

Our literature search identified only four published cases for PJI after TKA with a concomitant composite soft-tissue envelope and extensor apparatus deficiency. A limitation of our search is the lack of a precise definition of the extensor apparatus deficiency. The extent of tendon lesion does not linearly correlate with the clinical function of the knee joint. In our study, we narrowed the definition to that of the intraoperative judgement of the orthopaedic surgeon. Therefore, it is possible that our literature research missed publications with clinically inapparent extensor apparatus deficiencies. Three different reconstructive techniques were described: (i) a pedicled lateral gastrocnemius muscle with an attached lateral Achilles tendon, (ii) a pedicled MSAP gastrocnemius flap with a distally pedicled gracilis and semitendinosus tendon, and (iii) a free composite ALT flap that included vastus lateralis and FL 10, 16, 20, 21. The functional outcome reported was good at 12 and 18 months, and no other complications 16 apart from knee pain 20 were seen; instead, rather favourable outcomes 10, 21 were observed. In particular, recurrence of infection was denied, which in our series led to arthrodesis 1.8 and 2 years after plastic surgical reconstruction. The scarcity of literature for this growing clinical problem emphasizes the need for a structured orthoplastic treatment concept.

In conclusion, we presented a treatment concept based on a single-centre experience, which differentiated between various types of soft-tissue defects. We used a free sensate extended lateral arm flap with the triceps tendon, a free ALT flap with the fascia lata, or a pedicled MSAP gastrocnemius flap for soft-tissue reconstruction. This principle of reconstructing tissue 'like with like' provided promising functional and aesthetic results, as the replaced tissue contained intrinsic properties that were similar to those of the original tissue. Although we have outlined our rationale for this approach, we also acknowledge the concepts from other institutions 3 and the lack of data. Standardization of surgical techniques for a defined PJI problem are needed as is a consensus on study variables. We are convinced that this will facilitate interinstitutional comparisons of outcome data and therefore improvement of treatment concepts.

## Figures and Tables

**Figure 7:**
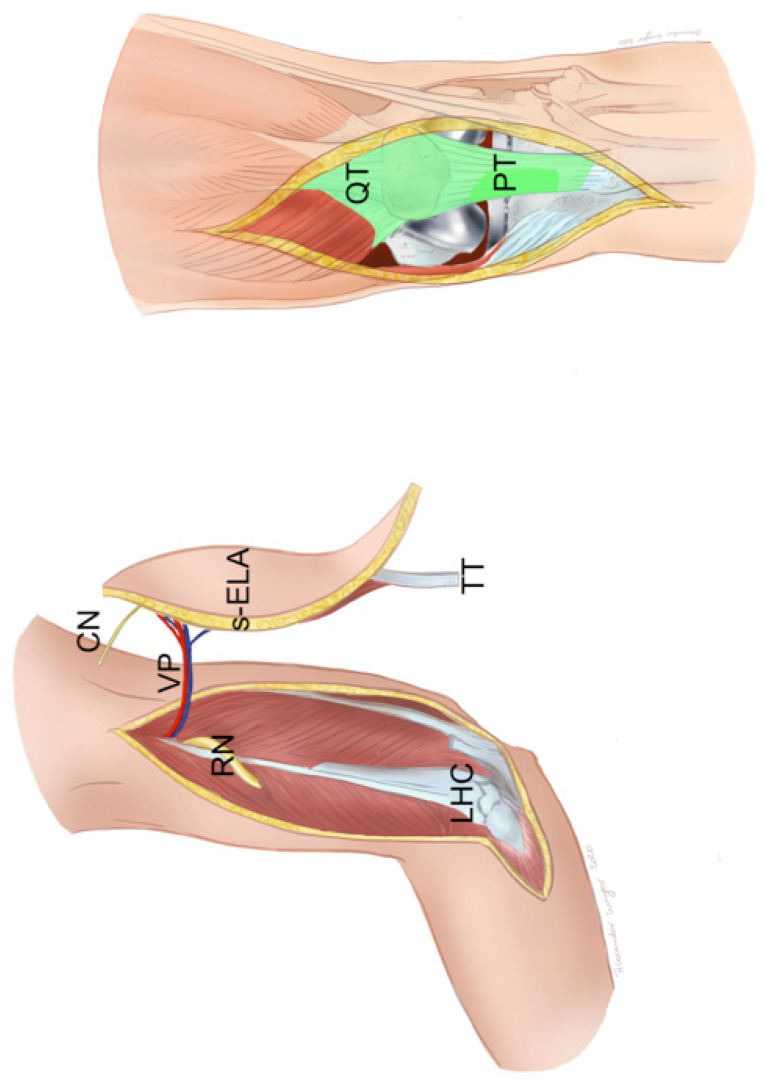
Free sensate extended lateral arm flap (s-ELA): Clinical scenario of PJI after TKA with a soft-tissue defect of up to 7 cm in width and an underlying extensor apparatus deficiency with either a partial patellar tendon (PT) lesion or partial quadriceps tendon (QT) lesion (light green). The s-ELA is raised with a strip of triceps tendon (TT) and part of the lateral head of the triceps muscle. At the proximal end of the flap can be seen the inferior lateral cutaneous nerve (CN) of the arm, which is raised with the flap in order to generate a sensate flap. Of note, the longer the vascular pedicle (VP) is dissected, the closer it lies to the radial nerve (RN), which is prone to neurapraxia. LHC: lateral humeral condyle (Table 1: Patient No. 3; additional Figures 5, 8, 9, 18, 19).

**Figure 10:**
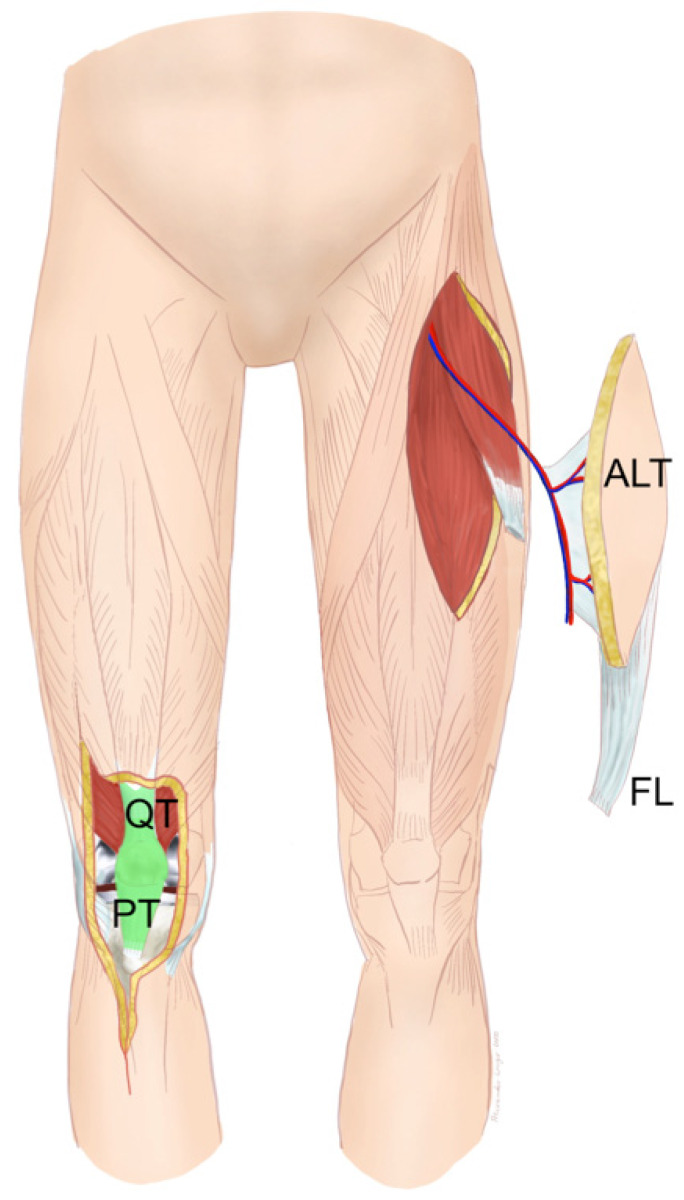
Free anterolateral thigh (ALT) flap with fascia lata (FL): Clinical scenario of PJI after TKA with a large soft-tissue defect of up to 10 cm in width and an underlying extensor apparatus deficiency with complete loss of the patellar tendon (PT), quadriceps tendon (QT), or a combination of both (green). The ALT flap is raised off the contralateral leg with the vascularized FL to reconstruct the damaged contralateral extensor apparatus (Table 1: Patient No. 1; additional Figures 3, 11).

**Table 1:** Orthoplastic treatment and outcome.

	Patient #				
	No. 1	No. 2	No. 3	No. 4	No. 5
**Figures**	3, 10, 11	4	5, 7, 8, 9, 18, 19		6, 12, 13, 14, 15, 16, 17
**Surgical data**					
Previous operations of the affected knee	9	3	20	5	3
Extent of extensor apparatus damage	50% PT 40% QT	100% PT 100% Patella 100% QT	70% PT	70% PT	40% PT
Plastic surgical technique	Free ALT+FL	Pedicled MSAP gastroc	Free s-ELA+TT	Free ALT+FL	Pedicled MSAP gastroc
**Outcome**					
1-year function		*			†
AROM [extension/flexion]	0/0/80°	*	0/0/100°	0/5/100°	0/0/90°
Follow-up [years]	2.75	*	8	6	0.17 ††
AROM [extension/flexion]	No	Unknown	0/0/100°	No	0/0/90°
Arthrodesis [years after reconstruction]	2.0	Unknown	No	1.8	No

PT: patellar tendon, QT: quadriceps tendon, ALT+FL: anterolateral thigh flap with fascia lata, MSAP gastroc: medial sural artery perforator gastrocnemius flap, s-ELA+TT: sensate extended lateral arm flap with triceps tendon, AROM: active range of motion. * No 1-year follow-up available, as was lost to follow-up. † No 1-year follow-up available, as operation was 2 months ago. Function described occurred at 2-month follow-up. †† Follow-up time 2 months.
